# *Lathyrus sativus* transcriptome resistance response to *Ascochyta lathyri* investigated by deepSuperSAGE analysis

**DOI:** 10.3389/fpls.2015.00178

**Published:** 2015-03-20

**Authors:** Nuno F. Almeida, Nicolas Krezdorn, Björn Rotter, Peter Winter, Diego Rubiales, Maria C. Vaz Patto

**Affiliations:** ^1^Instituto de Tecnologia Química e Biológica António Xavier, ITQB, Universidade Nova de LisboaOeiras, Portugal; ^2^GenXPro GmbHFrankfurt am Main, Germany; ^3^Institute for Sustainable Agriculture, Consejo Superior de Investigaciones CientíficasCórdoba, Spain

**Keywords:** ascochyta, grass pea, *Lathyrus sativus*, SuperSAGE, transcriptome, gene expression, resistance, candidate genes

## Abstract

*Lathyrus sativus* (grass pea) is a temperate grain legume crop with a great potential for expansion in dry areas or zones that are becoming more drought-prone. It is also recognized as a potential source of resistance to several important diseases in legumes, such as ascochyta blight. Nevertheless, the lack of detailed genomic and/or transcriptomic information hampers further exploitation of grass pea resistance-related genes in precision breeding. To elucidate the pathways differentially regulated during ascochyta-grass pea interaction and to identify resistance candidate genes, we compared the early response of the leaf gene expression profile of a resistant *L. sativus* genotype to *Ascochyta lathyri* infection with a non-inoculated control sample from the same genotype employing deepSuperSAGE. This analysis generated 14.387 UniTags of which 95.7% mapped to a reference grass pea/rust interaction transcriptome. From the total mapped UniTags, 738 were significantly differentially expressed between control and inoculated leaves. The results indicate that several gene classes acting in different phases of the plant/pathogen interaction are involved in the *L. sativus* response to *A. lathyri* infection. Most notably a clear up-regulation of defense-related genes involved in and/or regulated by the ethylene pathway was observed. There was also evidence of alterations in cell wall metabolism indicated by overexpression of cellulose synthase and lignin biosynthesis genes. This first genome-wide overview of the gene expression profile of the *L. sativus* response to ascochyta infection delivered a valuable set of candidate resistance genes for future use in precision breeding.

## Introduction

*Lathyrus sativus* (grass pea) is a diploid species (2n = 14; genome size of approximately 8.2 Gb, Bennett and Leitch, 2012) with a great potential for expansion in dry areas or zones that are becoming more drought-prone (Hillocks and Maruthi, 2012). This species has been also recognized as a potential source of resistance to several important diseases in legumes (Vaz Patto and Rubiales, 2014).

Ascochyta blights are among the most important plant diseases worldwide (Rubiales and Fondevilla, 2012). Among the legume species, ascochyta blights are incited by different pathogens. For example, ascochytoses are caused by *Ascochyta rabiei* (teleomorph *Didymella rabiei*) in chickpea, *A. fabae* (teleomorph *D. fabae*) in faba bean and *A. lentis* (teleomorph *D. lentis*) in lentil (Tivoli et al., 2006). Ascochyta blight in pea (*Pisum sativum*) is caused by a fungal complex formed by *A. pisi*, *A. pinodes* [teleomorph *Didymella pinodes* (syn. *Mycosphaerella pinodes*)] and *Phoma medicaginis* var. *pinodella* (Jones, 1927). Of these, *D. pinodes* is the most frequent and damaging (Tivoli and Banniza, 2007).

*Lathyrus* spp. (*L. sativus*, *L. cicera*, *L. ochrus*, and *L. clymenum*) however, are significantly more resistant to *D. pinodes* than field pea cultivars (Gurung et al., 2002). A detailed analysis of quantitative resistance of *L. sativus* to ascochyta blight, caused by *D. pinodes*, suggested that resistance in *L. sativus* may be controlled by two independently segregating genes, operating in a complementary epistatic manner (Skiba et al., 2004b). In another study, Skiba et al. (2004a) developed a grass pea linkage map and used it to locate two quantitative trait loci (QTL), explaining 12 and 9% of the observed variation in resistance to *D. pinodes*. Nevertheless, no candidate genes were identified at that time for these resistance QTLs, hampering their use in precision breeding. In an attempt to identify defense-related candidate genes involved in *D. pinodes* resistance in *L. sativus*, the expression of 29 potentially defense-related ESTs was compared between *L. sativus* resistant and susceptible lines (Skiba et al., 2005). These ESTs were selected from a previously developed cDNA library of *L. sativus* stem and leaf tissue challenged with *D. pinodes*. From these, 16 ESTs were considered eventually important for conferring stem resistance to ascochyta blight in *L. sativus*. In addition, the marker developed from one of them, EST LS0574 (Cf-9 resistance gene cluster), was significantly linked to one of the previously identified resistance QTLs. However, this study was necessarily limited to the small number of initially selected EST sequences.

deepSuperSAGE (Matsumura et al., 2012) is the combination of SuperSAGE (Matsumura et al., 2003) with high-throughput sequencing technologies, allowing genome-wide and quantitative gene expression profiling. Two recent studies applied this technique for the identification of genes involved in resistance to ascochyta blight in pea (Fondevilla et al., 2014) and faba bean (Madrid et al., 2013).

In the present study we employed deepSuperSAGE to obtain a genome-wide overview of the response of the transcriptome of a resistant *L. sativus* genotype to *A. lathyri* infection in comparison to a non-inoculated control. Thereby we aimed at elucidation of signaling pathways responding to *A. lathyri* infection and identification of candidate genes associated with resistance to ascochyta blight in grass pea as first step toward the development of effective strategies for legume resistance breeding against this pathogen.

## Materials and methods

### Plant material and inoculation

*Lathyrus sativus* genotype BGE015746, previously characterized by our team as resistant to *A. lathyri* (isolate “Asc.8”), not developing macroscopic disease symptoms (pers. comm.), was used for the experiments. Isolate “Asc.8” belongs to the fungal collection of the Institute for Sustainable Agriculture-CSIC (Córdoba, Spain) while the *L. sativus* genotype BGE015746 was kindly provided by the Plant Genetic Resources Centre (CRF-INIA), Madrid, Spain. Fifteen-days old seedlings, grown in plastic pots containing 250 cm^3^ of 1:1 sand-peat mixture in a controlled growth chamber (20 ± 2°C with a 12 h light photoperiod), were inoculated with the monoconidial *A. lathyri* isolate “Asc.8,” collected in Zafra, Spain. Three individual plants were used for each treatment (inoculated/control). Spore suspension for inoculation was prepared at a concentration of 5 × 10^5^ spores per milliliter and sprayed onto the plants' aerial parts as described by Fondevilla et al. (2014). Inoculated and control plants were then kept in the dark for 24 h at 20°C and with 100% relative humidity in order to promote spore germination and were then transferred to the initial growth chamber conditions. Resistance was confirmed by the absence of disease symptoms 15 days after inoculation (d.a.i.), while other *Lathyrus* spp. genotypes presented diverse levels of infection, ranging up to 60% of leaf area covered by lesions (pers. comm.).

### RNA extraction and deepsupersage library construction

Leaves from one plant per treatment were harvested at 2 h time intervals during the first 24 h after inoculation (h.a.i.). A total of 12 leaf samples per plant (one per each 2 h time point) were immediately frozen in liquid nitrogen after harvest and stored at −80°C. Total RNA was isolated from each sample separately, using the GeneJet Plant purification kit (Thermo Scientific, Vilnius, Lithuania) according to the manufacture's protocols. Isolated RNA was subsequently treated with Turbo DNase I (Ambion, Austin, TX, USA), and quantified by NanoDrop (Thermo Scientific, Passau, Germany). Hundred μg-samples of individual plant RNA from each time point were then pooled in two bulks, a control and an inoculated pool. RNA integrity was controlled by electrophoresis on a 2% agarose gel (Lonza, Rockland, USA) with SYBRSafe (Invitrogen, Eugene, USA) staining and visualized using a GEL-DOC 1000 System (Bio-Rad, Hercules, USA). deepSuperSAGE libraries from the two pools of control and inoculated RNAs were generated at GenXPro GmbH as described by Zawada et al. (2011). High-throughput DNA sequencing was performed on an Illumina Genome Analyser IIx using the Chrysalis 36 cycles v 4.0 sequencing kit. The multiplexed sequencing run consists of 38 sequencing cycles on a single lane.

### Data analysis and annotation

The sequence reads obtained by Illumina sequencing from each of the two pooled samples were processed with GenXPro's in-house analysis pipeline. Briefly, libraries were sorted according to their respective index, followed by elimination of PCR-derived tags identified by TrueQuant technology. The sequences representing distinct deepSuperSAGE tags were quantified. These unique sequences (UniTags) were subsequently annotated against various databases via BLAST (Altschul et al., 1990). A multi-step BLAST procedure was used to annotate the UniTag reads to ensure an unambiguous assignment to their corresponding transcript and to eliminate any remaining adaptor sequences. Reference datasets were generated by own de-novo-assembly (Almeida et al., 2014) and downloaded from the publicly accessible Fabaceae databases using the nucleotide database from the National Center for Biotechnology Information (NCBI). UniTag reads were successively aligned against these reference datasets in the following order: (1) 26 bp de-novo-assembly dataset with a minimum BLAST-score of 42; (2) UniTags which did not attain the specified BLAST score in the previous step were aligned against the complete NCBI dataset with the same required BLAST score of 42 or above. For each library, UniTag read numbers were normalized to a million sequenced reads in total (tags per million; TPM) to allow the comparison between the two (control/inoculated) libraries. *P*-values for the UniTags were calculated using a perl module (“http://search.cpan.org/~scottzed/Bio-SAGE-Comparison-1.00/”) (Velculescu et al., 1995; Audic and Claverie, 1997; Saha et al., 2002). The fold changes were calculated as the log2 ratio of the normalized values between the two libraries.

### Quantitative RT-PCR assay

For the quantitative RT-PCR assay, RNA samples from the different time points were pooled into two composite samples per plant, one control and one inoculated, in equimolar amounts. One μg of total RNA from each of these six composite samples (three plants/ two treatments) was reverse transcribed using the High Capacity cDNA Reverse Transcription Kit (Applied Biosystems, Foster City, CA, USA), according to manufacturer's instructions. For all studied genes, the product of each of these reactions was analyzed in technical duplicates, in a total of six technical replicates per treatment (inoculated/control). Analyzed genes were selected by their level of expression and tag count from the deepSuperSAGE analysis. The chosen UniTags differed between inoculated and control samples by log2 ratios ranging from −1.73 to 3.37, with UniTag counts ranging from 1 to 558. Primers were designed using the Primer3 software (Untergasser et al., 2012) (Table 1), and qRT-PCR reactions performed with an iQ™5 Real-Time PCR Detection System (Bio-Rad, Munich, Germany). Data analysis was performed using the Genex software package (MultiD, Goteborg, Sweden), by the geNorm software (Vandesompele et al., 2002).

**Table 1:** **Contig information and primer sequences for qRT-PCR**.

**Contig**	**BLAST hit**	**Forward primer 5′→3′**	**Reverse primer 5′→3′**
a7162;272	1-acyl-sn-glycerol-3-phosphate acyltransferase 1, chloroplastic-like	CATGCTTTGCTTCGGTGGAC	CTTGGACCGCCATTGCAATC
a600;793	40S ribosomal protein S24	GCGGACAAGGCAGTCACTAT	GGCCTTTGAGACATTAGCCCT
a574;578	40S ribosomal protein S29	ATGGACTCATGTGCTGCAGG	AAACCTAACCTTGGCTGGCC
a11456;203	ABC transporter A family member 1-like	TGCATCCATCATGGTGACGG	TGCTGCCCAGTTTCACTGTT
a14590;204	Abhydrolase domain containing protein	CCCGACAGTGAATCCCTTCC	ACAGACAGCAGTGCCGAAAT
a6507;507	β-tubulin	TGCCTAGGATCAGCAGCACA	TCAGTGTCCCTGAGCTCACT
a5354;161	Histone H3	ACGCTCGCCTCTAATACGC	GCAGCTGAGTCGTACCTTGT
a833;622	L-allo-threonine aldolase	AGTCACGGAATCACCAAATCCC	ATCGTCTCGTGGCTTGTGG
a6396;394	Malic enzyme	TTGGCTACGCATCTTCCTCG	GCTTCTGTTCACCTATAGTTGCGG
a156;1828	Oxygen-evolving enhancer protein 3 precursor	GTACTTCTCTGCTTCTGAGGGAC	CCAAGCCTAAGGACCAGAAACA
a8658;358	Primary amine oxidase-like	GGGCCTTTCAAAGCTTGGC	TGTTCCTCCAAGCCCAAGTG
a18319;129	RNA polymerase II C-terminal domain phosphatase-like protein	AATCTCGCGATCCACGTCAC	TGGCTTGTGGAACGAATGAGG
a1255;508	Unknown	AGTGCGGGTATGGAATCACG	TGGGACACCAGATGAATGGC
a10868;260	Villin-4	GTCAGCTCCCGGCAGTTTAG	AAAGTTTCCCGGGAGCAGTC

### UniTag assignment to functional categories

In order to classify the *L. sativus* UniTags into functional categories, the Mercator pipeline for automated sequence annotation (Lohse et al., 2014), available at http://mapman.gabipd.org/web/guest/app/mercator, was employed. The mapping file was created using only significantly (−1 ≤ log2 fold change ≥1; *p*-value < 0.05) up- and down-regulated UniTags and accessing the following, manually curated databases: *Arabidopsis* TAIR proteins (release 10), SwissProt/UniProt Plant Proteins (PPAP), TIGR5 rice proteins (ORYZA), Clusters of orthologous eukaryotic genes database (KOG), Conserved domain database (CCD) and InterPro scan (IPR). The Mercator mapping file was then employed for analysis by the MapMan software (Thimm et al., 2004), available at http://mapman.gabipd.org/web/guest/mapman.

## Results

### SuperSAGE library characterization

A total of 399,648 deepSuperSAGE 26 bp-tags were obtained. Of these 205,691 tags were derived from *L. sativus* inoculated with *A. lathyri* and 193,957 tags from control plants. These tags corresponded to 14,386 unique sequences (UniTags) of which 13,773 (95.7%) were successfully annotated to the *L. sativus* reference dataset (Almeida et al., 2014).

When comparing inoculated versus control samples, 738 UniTags were differentially expressed (DE) [log2 fold ≥2 (up) or log2 fold ≤ −2 (down); *p*-value < 0.05]. Of the differentially expressed UniTags, 625 (84.7%) were successfully annotated in public plant databases. 354 UniTags matched also to entries in fungal databases, but annotation scores were always lower than the plant database hit, and therefore were considered UniTags of plant origin. From the 625 differentially expressed UniTags with BLAST hit, 382 (61.1%) were up-regulated while 243 (38.9%) were down-regulated. The full list of differentially expressed UniTags can be found in Supplementary file 1.

### SuperSAGE validation by quantitative RT-PCR assay

**Figure 1:**
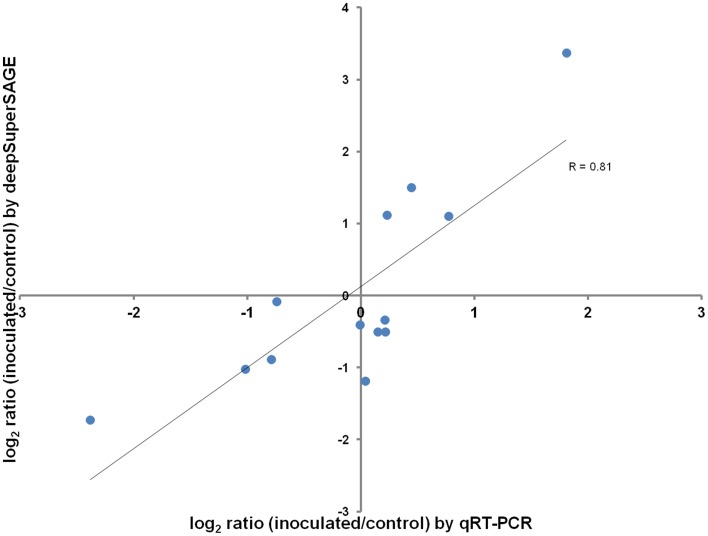
**Relative expression levels correlation between RNA-seq and qRT-PCR**. Pearson's correlation coefficient (R) between relative expression levels is shown below the trend line.

From the geNorm software analysis the best housekeeping gene for the quantitative RT-PCR validation was “β-tubulin” (transcript a6507;507). The expression levels of the remaining 13 genes analyzed by qRT-PCR to validate the RNA-seq results are present in Table 2. A good correlation (*R* = 0.8) was observed between the log2 fold changes measured by deepSuperSAGE and qRT-PCR for the genes tested (Figure 1).

**Table 2:** **Log2 fold expression results for deepSuperSAGE and qRT-PCR experiments**.

**Contig**	**BLAST hit**	**BGE015746**					
		**Control tag counts**	**Control tags per million**	**Inoculated tag counts**	**Inoculated tags per million**	**Inoculated/control deep SuperSAGE (log2)**	**Inoculated/control qPCR (log2)**
a7162;272	1-acyl-sn-glycerol-3-phosphate acyltransferase 1, chloroplastic-like	1	51,558	11	534,783	3.375	1.81
a600;793	40S ribosomal protein S24	76	391,839	57	277,115	−0.500	0.15
a574;578	40S ribosomal protein S29	171	881,639	128	622,293	−0.503	0.21
a11456;203	ABC transporter A family member 1-like	10	515,578	23	111,818	1.117	0.23
a14590;204	Abhydrolase domain containing protein	5	257,789	4	194,466	−0.407	−0.01
a5354;161	Histone H3	54	278,412	31	150,712	−0.885	−0.79
a833;622	L-allo-threonine aldolase	12	618,694	36	17,502	1.500	0.45
a6396;394	Malic enzyme	18	928,041	41	199,328	1.103	0.77
a156;1828	Oxygen-evolving enhancer protein 3 precursor	558	287,693	292	141,961	−1.019	−1.02
a8658;358	Primary amine oxidase-like	122	629,005	39	189,605	−1.730	−2.38
a18319;129	RNA polymerase II C-terminal domain phosphatase-like protein	31	159,829	26	126,403	−0.338	0.21
a1255;508	Unknown	36	185,608	36	175,02	−0.085	−0.74
a10868;260	Villin-4	30	154,673	14	680,633	−1.184	0.04

### Annotation of differentially expressed genes in the resistant *L. sativus* genotype after *A. lathyri* infection

**Figure 2:**
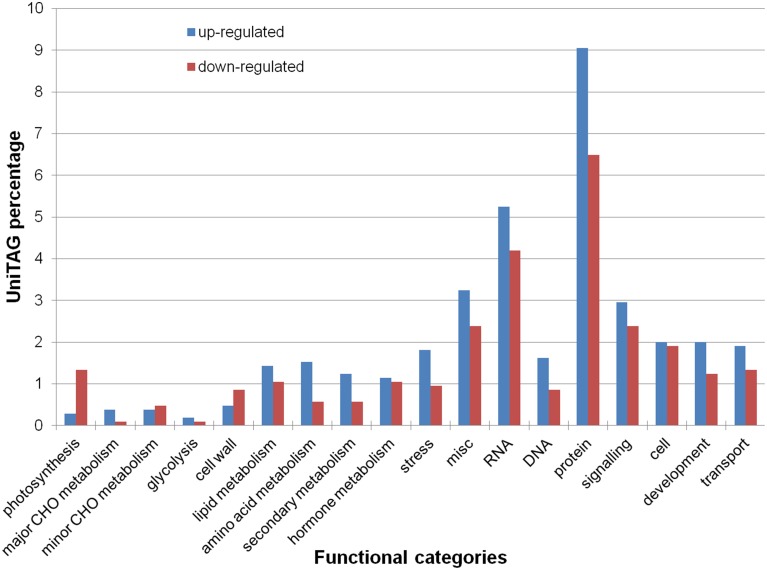
**Percentage of annotated up- and down-regulated *L. sativus* UniTags upon *A. lathyri* inoculation in several functional categories by MapMan**.

Functional annotation of the UniTags via Mercator and MapMan, grouped 625 UniTags (382 up- and 243 down-regulated) into 25 main functional categories. Most represented categories from up-regulated UniTags were “protein metabolism” (11.6% up- and 8.1% down-regulated), “RNA metabolism” (9.4% up- and 4.7% down-regulated), “miscellaneous” (5.7% up- and 3.4% down-regulated), “signaling” (4.7% up- and 3.4% down-regulated) and “cell metabolism” (4.2% up- and 2.7% down-regulated) (Figure 2).

Potential candidate genes assigned to stress-related functional categories are listed in Table 3.

**Table 3:** **List of detected genes by functional category, expression values and putative function as described in Mercator**.

**Functional category**	**Up/Down- regulated**	**Gene**	**Contig**	**log2 fold change**	**Putative function**
**Stress**		**Total of 13 genes**			
Biotic	Up	Multidrug and toxin compound extrusion (MATE) efflux	a10578;232	10.1	Transport
		Armadillo (ARM) repeat superfamily protein	a11957;197	9.2	Protein degradation
		Acidic endochitinase precursor (E.C. 3.2.1.14)	a3844;425	2.7	Antimicrobial activity
		RESISTANCE TO *P. SYRINGAE* PV MACULICOLA 1 (RPM1)	a15229;117	2.7	Pathogen recognition
		PR-1-like protein	a8364;304	2.5	Pathogenesis related - function unknown
		Disease resistance-responsive (dirigent-like protein)	a19559;148	2.4	Pathogenesis related - function unknown
	Down	Similar to a chitin-binding protein (PR-4)	a4526;396	−2.7	Antimicrobial activity
Abiotic	Up	DNAJ heat shock protein	a14646;184	9.2	Protein folding
		DNAJ heat shock protein	a11774;196	2.9	Protein folding
		Heat shock protein 101 family	a22155;174	2.7	Thermotolerance to chloroplasts
	Down	S-adenosyl-L-methionine-dependent methyltransferases superfamily proteins	a76762;48	−1.0	Methylation
		S-adenosyl-L-methionine-dependent methyltransferases superfamily proteins	a15131;185	−9.3	Methylation
		Damaged DNA binding protein 1A	a7531;356	−2.4	Negative regulation of photomorphogenesis
**Secondary metabolism**		**Total of 8 genes**			
Flavonoids	Up	Chalcone reductase	a124260;32	9.6	Flavonoid biosynthesis
Isoprenoids	Up	Violaxanthin de-epoxidase	a1039;529	10.2	Isoprenoid biosynthesis
		Tocopherol cyclase	a708;558	9.8	Isoprenoid biosynthesis
		Beta-hydroxylase 1	a3019;480	9.2	Isoprenoid biosynthesis
		RAB geranylgeranyl transferase beta subunit 1	a18716;198	2.7	Isoprenoid biosynthesis
	Down	Pyridoxal phosphate (PLP)-dependent transferases superfamily protein	a23319;167	−9.3	Isoprenoid biosynthesis
Phenylpropanoids	Up	Phenylalanine ammonia-lyase 1 (PAL1)	a5118;361	3.1	Lignin biosynthesis
		4-coumarate-CoA ligase	a9524;336	2.4	Lignin biosynthesis
**Cell wall**		**Total of 9 genes**			
Precursor synthesis	Up	UDP-sugar pyrophospharylase	a18802;199	9.2	Cell wall synthesis
Cellulose synthesis	Up	IRREGULAR XYLEM 1 (IRX1)	a12901;208	2.9	Cell wall synthesis
	Down	Cellulose synthase isomer (CESA3)	a6154;437	−9.0	Cell wall synthesis
		Cellulose-synthase-like C5 (CSLC5)	a69762;64	−9.0	Cell wall synthesis
		Glycosylphosphatidylinositol-anchored protein COBRA-like (COB)	a5236;405	−2.2	Cell wall synthesis
**Cell wall**		**Total of 9 genes**			
Degradation	Down	β-xylosidase 1 (BXL1)	a4868;387	−2.5	Cell wall degradation
Modification	Down	Xyloglucan endotransglycosylase-related protein (XTR4)	a2002;437	−2.9	Cell wall modifications
Pectin^*^esterases	Up	SKU5 similar 9 (sks9)	a34641;119	9.4	Cell wall modifications
	Down	Plant invertase/pectin methylesterase inhibitor superfamily	a7441;320	−2.4	Cell wall modifications
**Hormone metabolism**		**Total of 6 genes**			
Ethylene	Up	Basic helix-loop-helix (bHLH) DNA-binding superfamily protein	a246012;14	9.8	Induced by ethylene
		1-aminocyclopropane-1-carboxylate synthase (ACC)	a11244;194	9.4	Ethylene biosynthesis
		Calmodulin-binding transcription activator protein with CG-1 and Ankyrin domains	a7309;224	8.9	Induced by ethylene
	Down	RING E3 ligase, XBAT32	a25116;120	−2.9	Inhibitor of ethylene biosynthesis
Salicylic acid	Up	UDP-glucosyltransferase 74F1	a25008;62	9.2	Salicylic acid biosynthesis
Abscisic acid	Up	Plasma membrane protein KOBITO (KOB1)	a7591;247	2.7	Abscisic acid signal transduction
**Miscellaneous**		**Total of 4 genes**			
Glutathione S transferases	Up	Glutathione S-transferase	a20761;129	9.6	Detoxification
Peroxidases	Down	Peroxidase superfamily protein	a20130;174	−9.3	Production of reactive oxygen species
Beta 1,3 glucan hydrolases	Up	Glucan endo-1,3-beta-glucosidase	a243608;22	8.9	Antimicrobial activity
	Down	Glucan endo-1,3-beta-glucosidase 11-like	a34766;162	−3.1	Antimicrobial activity

## Discussion

The present study provides the first comprehensive overview of gene expression of the *L. sativus* response to ascochyta infection. It delivered a valuable set of grass pea sequences for resistance candidate gene discovery and use in precision breeding for this species.

deepSuperSAGE analysis of a ascochyta blight resistant grass pea genotype, using control and inoculated plants, generated 14.387 UniTags. Of those, 95.7% mapped to a recently published reference grass pea/rust interaction transcriptome assembly (Almeida et al., 2014). From the total mapped UniTags, 738 were differentially expressed between control and inoculated conditions, 625 of which could be annotated in public plant databases.

Although differences may be observed between deepSuperSAGE and qRT-PCR results due to the presence of different transcript isoforms from the same gene, or different genes from the same family that cannot be distinguished by the 26-bp tag of the 3′-untranslated region provided by deepSuperSAGE (Fondevilla et al., 2014), the validation of 13 differentially expressed genes by RT-qPCR, using three biological replicates, provided a good correlation with deepSuperSAGE results. Interestingly, the most invariably expressed UniTag corresponded to a β-tubulin transcript. This transcript was also identified as the best normalization gene in a previous RNA-seq study, where this genotype (BGE015746) was inoculated with *Uromyces pisi* (Almeida et al., 2014).

The functional interpretation of differential gene expression patterns provided evidence for the involvement of genes assigned to several functional categories in different phases of the plant/pathogen interaction. As listed in Table 3, the most significant stress-related responses of the resistant genotype, however, were probably the clear-cut up-regulation of the ethylene signaling pathway represented by genes involved in ethylene synthesis and down-regulation of inhibitors of ethylene synthesis and the up-regulation of ethylene-induced genes. Another prominent response concerned alterations in the cell wall metabolism, as indicated by the up-regulation of cellulose synthase genes and genes related to lignin biosynthesis. Pathogenesis-related functions induced by ascochyta infection are discussed below.

### Pathogen perception

The first step in plant defense response is pathogen detection by pattern recognition receptors (PRR) as part of the innate immune system. This pathogen perception will trigger signaling events that activate a broad array of downstream defensive measures in the plant (Nicaise et al., 2009). In this study we identified several differentially expressed receptor kinases (up- and down-regulated) containing leucine rich repeats (LRRs), that are key players in the regulation of diverse biological processes such as development, hormone perception and/or plant defense (Torii, 2004). We also identified an up-regulated receptor kinase with a thaumatin-like domain (a36033;97, log2 fold = 9.4). Thaumatin is a pathogenesis related (PR) protein described as increasing the permeability of fungal membranes by pore-forming mechanisms and therefore restraining fungal growth or even killing it (Selitrennikoff, 2001). Several thaumatin-like proteins have been shown to increase resistance in potato (Acharya et al., 2013), rice (Datta et al., 1999), wheat (Anand et al., 2003), and grapevine (Jayasankar et al., 2003) to diverse fungal pathogens. Several transcription factors were also induced upon pathogen recognition. One “WRKY DNA-binding protein 4” (a8940;191, log2 fold = 8.9) was identified in our study as up-regulated after inoculation. WRKY transcription factors are induced after the recognition by intracellular receptors of pathogen virulence molecules (effectors). After its induction, WRKY transcription factors can positively or negatively regulate various aspects of pathogen-associated molecular pattern (PAMP)-triggered immunity (PTI) and effector triggered immunity (ETI) (review by Eulgem, 2005). Also related to ETI, we found an up-regulated transcript with homology to *Arabidopsis* “RESISTANCE TO *P. SYRINGAE* PV MACULICOLA 1 (RPM1),” known to confer resistance to *Pseudomonas syringae* strains containing the avirulence genes *avrB* and *avrRpm1* (Bisgrove et al., 1994). In the incompatible interaction in the model plant, RIN4 (RPM1 interacting protein 4) interacts with RPM1, to prevent its activation. Reduction of RIN4 expression enhances resistance to *P. syringae* and to the oomycete *Paranospora parasitica*. Therefore RIN4 is considered a negative regulator of basal plant defenses that is activated by *P. syringae*'s *avrB* and *avrRpm1* (Mackey et al., 2002). Assuming a similar function of the RPM1-homolog in grass pea-ascochyta interaction this gene could be a resistance-steering candidate gene. It would be further interesting to know whether up-regulation of the RPM1-homolog is part of a broad defense response, or if it is activated by a specific *Ascochyta* spp. effector that the grass pea's RPM1 is able to recognize.

### Hormone signaling

It is generally accepted that biotrophic pathogens usually trigger the salicylic acid (SA) pathway, while necrotrophic pathogens activate jasmonic acid (JA) and the ethylene (ET) pathways (Glazebrook, 2005; Bari and Jones, 2009). The nature of the initial phases of *Ascochyta* spp. infection in grass pea is still not completely understood. Normally considered as necrotroph, there is evidence, at least for some *Ascochyta* spp., for an early biotrophic phase spanning from the penetration of the epidermis of the plant until the initial colonization of the mesophyll (Tivoli and Banniza, 2007).

Our data, however, demonstrate that the ethylene pathway may have a major role in resistance of at least our grass pea accession to *A. lathyri*, in line with the necrotrophic nature of the interaction. For example, the “1-aminocyclopropane-1-carboxylate synthase (ACC)” gene involved in ET biosynthesis and other two genes described by Mercator (Lohse et al., 2014) as being induced by ethylene [“Calmodulin-binding transcription activator with CG-1 and Ankyrin domains” and “basic helix-loop-helix (bHLH) DNA-binding superfamily protein”] were significantly up-regulated upon infection. The transcript is homologous to the “Calmodulin-binding transcription activator with CG-1 and Ankyrin domains” previously identified as similar to “Calmodulin-binding protein/ER66 protein” from tomato (Skiba et al., 2005). It seems that in grass pea either different transcript isoforms or a gene family exists, since in Skiba et al. (2005), 16 defense-related ESTs were identified with a greater or/and earlier expression in stems of resistant *L. sativus* genotypes compared with susceptible ones upon ascochyta blight inoculation. In our study from those only “Calmodulin-binding transcription activator with CG-1 and Ankyrin domains” was up-regulated whereas three other SuperTags with similar annotation were not differentially expressed. The incongruence between our results and that of Skiba et al. (2005) may be explained by the different mechanism of resistance, since the *L. sativus* genotype used by Skiba et al. (2005), ATC 80878, is partially resistant, and the genotype used in our study, BGE015746, displays complete resistance. Furthermore, the pathogen isolates used in both studies were also different, since the ATC 80878 genotype was inoculated with a mixture of three highly aggressive (on several *P. sativum* genotypes) *M. pinodes* isolates (WAL3, T16, and 4.9) whereas our ascochyta inoculum was a monoconidial *A. lathyri* isolate.

Additionally in our study, “RING E3 ligase, XBAT32,” an ubiquitin described as negative regulator of ET biosynthesis in *Arabidopsis* during plant growth, development and salt stress (Prasad and Stone, 2010), was down-regulated again stressing the importance of ET for resistance in our *L. sativus* genotype. ET pathway induction was also observed by microarray and deepSuperSAGE analyses during the response of a resistant pea genotype to ascochyta blight infection (Fondevilla et al., 2011, 2014). Thus, up-regulation of ET signaling may be a general response of temperate legumes to ascochyta blight infection.

Although the ET pathway was the only hormone pathway clearly up-regulated, other genes involved in hormone signaling were also up-regulated. These included “UDP-glycosyltransferase 74 F1 (UGT74F1),” and “phenylalanine ammonia-lyase 1” (PAL1), both involved in SA biosynthesis in *Arabidopsis* (Mauch-Mani and Slusarenko, 1996).

### Cell wall fortification

*Ascochyta lathyri* penetrates the host's epidermal cells via an as yet unperfectly described biotrophic or necrotrophic phase to reach the mesophyll. However, it is known that during pathogen penetration, the plant's cell wall is not just a static physical barrier. The perception of cell wall degradation by the pathogen can activate local plant responses that trigger repair and fortification mechanisms via expression of different genes as e.g. the cell wall synthesis precursor, “UDP-sugar pyrophosphorylase” (Gibeaut, 2000) or the cellulose synthase “IRREGULAR XYLEM 1 (IRX1)” genes both involved in cell wall synthesis (Taylor et al., 2000). Both were up-regulated in our grass pea genotype after *A. lathyri* inoculation. IRX1 was also up-regulated in the same genotype BGE015746 in response to the infection with rust (Almeida et al., 2014), suggesting that the induction of this cellulose synthase, and consequently cell wall strengthening, may play an important role in resistances of this grass pea genotype to diverse pathogens. Improving the cell wall lignin content is another common plant defense mechanism. In our study, inoculation elicited the expression of three UniTags representing genes implicated in cell lignification: “4-coumarate-CoA ligase,” involved in lignin biosynthesis (Lee et al., 1995), “disease resistance-responsive (dirigent-like),” previously identified as improving lignin content at infection sites (Zhu et al., 2007) and a “phenylalanine ammonia-lyase 1 (PAL1),” previously related to SA biosynthesis and also to the synthesis of lignin precursors (Mauch-Mani and Slusarenko, 1996).

However, there were also cell wall synthesis genes that were down-regulated. For example, three transcripts involved in cellulose biosynthesis [“glycosylphosphatidylinositol-anchored protein COBRA-like (COB),” “cellulose synthase isomer (CESA3),” and “cellulose-synthase-like C5 (CSLC5)”] and two pectinesterase transcripts involved in cellulose biosynthesis and in cell wall modifications (Dai et al., 2011; Hansen et al., 2011; Liu et al., 2013), were down-regulated after inoculation. Though this is somehow unexpected in a resistant accession it may be explained by results from *Arabidopsis* where CESA3-deficient mutants reduced their cellulose synthesis, but instead activated lignin synthesis and defense responses through the jasmonate and the ethylene signaling pathways (Cano-Delgado et al., 2003; Hamann, 2012). These observations suggest that mechanisms monitoring cell wall integrity can activate lignification and defense responses. Therefore, cellulose biosynthesis may not only be involved in the first line of defense but also in signaling as an indirect defense mechanism. Histological analysis will allow clarifying this hypothesis in the future.

Additionally, “beta-xylosidase 1 (BXL1)” and “xyloglucan endotransglycosylase-related protein (XTR4),” were found down-regulated after inoculation. BXL1 is involved in development of normal (non-infected) cell walls. BXL1 deficient *Arabidopsis* mutants showed alterations of cell wall composition and in plant development (Goujon et al., 2003). XTR4 belongs to the xyloglucan endotransglycosylase gene family, the so called endoxyloglucan transferases, that are involved in hemicellulose metabolism. Interestingly, XTR4 is down-regulated in *Arabidopsis* by the growth hormone auxin (Xu et al., 1996). Therefore, in grass pea these genes may be down-regulated under the mechanisms regulating cell wall thickening to restrict fungal penetration.

Taken together these results hint to a general reshuffling of cell wall components that exchanges certain cellulose types, restricts hemicelluloses and favors lignin as part of the resistance reaction of a resistant *L. sativus* genotype. To which extent these mechanisms contribute to resistance needs to be determined in populations segregating for resistance.

### Antimicrobial activity

Upon infection plants increase the production of antibacterial defense proteins to limit colonization by the pathogen (Consonni et al., 2009). After inoculation of grass pea with *A. lathyri*, “chitinase A (PR-3)” was up-regulated. Chitinases are involved in the inhibition of fungal hyphae growth in intercellular spaces as a defense response to fungal infection in several plant species (reviewed by Grover, 2012). Additionally, a “GDSL lipase 1,” another antimicrobial compound that also functions as ET-dependent elicitor (Kwon et al., 2009), and a “pathogenesis-related protein (PR-1-like)” with antifungal properties (Van Loon and Van Strien, 1999) were up regulated. This PR-1-like transcript is similar to an EST sequence (DY396405) identified previously in the response of grass pea to *M. pinodes* (Skiba et al., 2005), but in that study it showed low to mid-level expression in leaf and stem tissue, with little difference between resistant and susceptible genotypes. PR-1-like genes were also up-regulated in the resistance response of our grass pea accession BGE015746 to rust infection (Almeida et al., 2014). Chitinases were also found up-regulated in the resistance response of pea to ascochyta blight infection (Fondevilla et al., 2014).

The phenylpropanoid secondary metabolite biosynthesis pathway is notorious for the production of antimicrobial compounds in plants. In our resistant genotype, inoculation elicited a “chalcone reductase” transcript coding for an enzyme that co-acts with chalcone synthase in the first step of flavonoid biosynthesis (Naoumkina et al., 2010). Interestingly, in a previous study in *L. sativus* a chalcone reductase EST was also up-regulated as a defense reaction after inoculation with *M. pinodes* (Skiba et al., 2005).

### Reactive oxygen species

Reactive oxygen species (ROS) in plants are generated normally as by-products of oxidative phosphorylation and diverse biosynthetic pathways. Under non-stress conditions these potentially deleterious molecules are controlled by antioxidants. Under biotic or abiotic stress however, ROS production increases as part of the anti-microbial response. Their rapid accumulation of ROS creates an oxidative burst that may induce cell death and restricts the establishment of the pathogen in the plant (Apel and Hirt, 2004). In our study however, the lack of visual symptoms of a hypersensitive response or necrosis in the inoculated resistant grass pea, suggests that the over-production of ROS is not important for resistance in this plant/pathogen interaction. Moreover, our transcriptomic data reflects this aspect, since the only differentially expressed UniTag related to ROS regulation was a “peroxidase” which was down-regulated after inoculation.

### Detoxification

During defense response, plants produce toxic compounds for defense and are themselves attacked by toxins secreted by the pathogen. To cope with toxins from the pathogen, plants developed several detoxification mechanisms. In our grass pea accession two UniTags related to detoxification were up-regulated upon *A. lathyri* infection, namely a “phytoene synthase,” a precursor in the carotenoids biosynthesis pathway and a “glutathione S-transferase (GST).” Carotenoids are lipophilic antioxidants being able to detoxify various forms of ROS, playing an important role in both biotic and abiotic stress responses (Young, 1991; Ramel et al., 2012). GSTs form a large family of enzymes that have diverse roles in detoxifying xenobiotics, antioxidant activity, or ROS scavenging (Dalton et al., 2009). ROS scavengers are needed to maintain ROS activity levels below the oxidative damage threshold (Moller et al., 2007). GST was also found up-regulated upon inoculation in an ascochyta blight resistant pea genotype challenged with *M. pinodes* (Fondevilla et al., 2014), corroborating its important role in resistance.

## Conclusions

Our deepSuperSAGE analysis provided deep insights into the molecular mechanisms underlying resistance to *A. lathyri* in *L. sativus* suggesting candidate genes and pathways potentially involved in ascochyta blight resistance in a particular, completely resistant genotype. Resistance reactions involved a wide range of reactions including changes in hormone signaling, biotic and abiotic stress reactions, cell wall metabolism and in the secondary metabolism that can now be further investigated. In particular, this study suggests a strong up-regulation of the ET pathway and of cell wall fortification upon inoculation with *A. lathyri*. In agreement with the macroscopic phenotypic observations 15 d.a.i., that gave no hint to the presence of an oxidative burst or hypersensitive response, the changes in transcripts related to ROS management were rather moderate. Thus, we conclude that the resistance of our *L. sativus* genotype BGE015746 to ascochyta is quantitative rather than qualitative, as it has been reported in other legume species such as pea (Carrillo et al., 2013), lentil (Tullu et al., 2006), faba bean (Rubiales et al., 2012), and chickpea (Hamwieh et al., 2013) and represents a potentially lasting source of resistance to ascochyta blight (Rubiales et al., 2015). To exploit this genotype for resistance breeding next steps will include, on one hand, the identification of polymorphisms in the identified candidate resistance genes to facilitate resistance breeding by marker-assisted selection. On the other hand, we will use histological approaches to characterize in detail the type of resistance response and correlate it with the molecular mechanisms identified in this study. A deeper understanding of resistance mechanism and facilitated resistance breeding will help to harness grass pea for agronomy in dry areas or zones that are becoming more drought-prone due to global climate change in the future.

### Conflict of interest statement

The authors declare that the research was conducted in the absence of any commercial or financial relationships that could be construed as a potential conflict of interest.
